# Association of Life-Course Educational Attainment and Breast Cancer Grade in the MEND Study

**DOI:** 10.5334/aogh.3142

**Published:** 2021-07-07

**Authors:** Anjali Gupta, Kelley Jones, April Deveaux, Malcolm Bevel, Omolola Salako, Adetola Daramola, Allison Hall, Olusegun Alatise, Gabriel Ogun, Adewale Adeniyi, Akinlolu Ojo, Omobolaji Ayandipo, Thomas Olajide, Olalekan Olasehinde, Olukayode Arowolo, Adewale Adisa, Oludolapo Afuwape, Aralola Olusanya, Aderemi Adegoke, Trygve O. Tollefsbol, Donna Arnett, Christopher B. Newgard, Tomi Akinyemiju

**Affiliations:** 1 Trinity College of Arts and Sciences, Duke University, Durham, NC, USA; 2 Department of Population Health Sciences, School of Medicine, Duke University, Durham, NC, USA; 3 College of Medicine & Lagos University Teaching Hospital, University of Lagos, Lagos State, Nigeria; 4 Department of Pathology, School of Medicine, Duke University, Durham, NC, USA; 5 Obafemi Awolowo University Teaching Hospital, Ile-Ife, Osun State, Nigeria; 6 Unversity College Hospital, University of Ibadan, Ibadan, Oyo State, Nigeria; 7 Federal Medical Center, Abeokuta, Ogun State, Nigeria; 8 University of Kansas Medical Center, Kansas City, KS, USA; 9 Our Lady of Apostle Catholic Hospital, Ibadan, Oyo State, Nigeria; 10 University of Alabama at Birmingham, Birmingham, AL, USA; 11 University of Kentucky, Lexington, KY, USA; 12 Duke Cancer Institute, School of Medicine, Duke University, Durham, NC, USA; 13 Duke Global Health Institute, Duke University, NC, USA

## Abstract

**Background:**

Nigeria reports the highest age-standardized mortality rate for breast cancer (BC) among African countries and disproportionately high rates of high-grade cancer. Histological grade is a strong predictor of mortality, and evidence suggests that educational attainment influences cancer outcomes.

**Objective:**

We characterize the association between educational trends across the life-course and BC grade at diagnosis.

**Methods:**

Data on 224 BC patients enrolled in the Mechanisms for Established and Novel Risk Factors for Breast Cancer in Nigerian Women (MEND) study was analyzed. Participant and parental (mother and father) education was categorized as low (primary school or less) or high (secondary school or greater). Accordingly, the educational trend across the life-course was determined for each participant relative to each parent: stable high, increasing, decreasing, or stable low. BC grade was classified as high (grade 3) or low (grades 1–2).

**Findings:**

About 34% of participants, 71% of fathers, and 85% of mothers had low education. Approximately one-third of participants were diagnosed with high-grade BC. Participants with low-grade BC were more likely to have highly educated fathers (p = 0.04). After adjusting for age, comorbidities, marital status and mammogram screening, participants with highly educated fathers were 60% less likely to have high-grade BC (aOR 0.41; 95% CI 0.20 to 0.84) compared to those with less-educated fathers. Stable high life-course education relative to father was also associated with a significantly lower likelihood of having high-grade BC (aOR 0.36; 95% CI 0.15 to 0.87) compared to stable low life-course education. No significant associations were observed for the participant’s education, mother’s education, or life-course education relative to mother.

**Conclusions:**

Early-life socioeconomic status (SES) may influence BC grade. This deserves further study to inform policies that may be useful in reducing high-grade BC in Nigeria.

## Introduction

Breast cancer (BC) poses a global challenge, with an estimated 2.1 million cases diagnosed and over 0.6 million deaths in 2018 [1]. BC is the leading cause of cancer death among females [1]. According to data from 2010, women of reproductive age in developing countries are twice as likely to develop BC than their counterparts in developed countries, where cases typically occur in older women [2]. In addition, while BC incidence in Africa is lower than in all other continents except Asia, age-standardized mortality rates rank the highest globally. Nigeria, the most populous African nation, suffers from the highest mortality rate of BC among African countries [3].

Histological BC grade has significant prognostic value independent of cancer stage, with low-grade cancers associated with improved survival relative to high-grade cancers [4,5]. Studies in Nigeria have reported a worrisome prevalence of high-grade BC, with disproportionately high numbers of aggressive cancer subtypes or clinical course when compared to White populations [6,7]. Understanding how critical socioeconomic factors, such as education, influence BC grade is crucial to developing effective interventions and policies that can improve negative BC outcomes. In general, higher literacy is associated with improved health outcomes [8], an association that is likely driven by greater work opportunities, and access to resources useful for maintaining health [9]. There is strong evidence that educational attainment significantly influences cancer mortality [10]. It is associated with earlier BC stage at diagnosis [11,12,13,14], and increased access to BC screening [15,16]. Research regarding the relationship between education and BC grade, specifically, is very limited and conflicting. In England, one study found that socioeconomic deprivation is associated with higher BC grade at diagnosis [17]. However, a study in a Turkish population reported no significant association between education level and BC grade [18]. Further research is needed to better characterize this relationship.

In addition to individual educational attainment, parental education level and educational trends across the life-course may also impact BC outcomes. A recent systematic review suggests that early-life socioeconomic status (SES) may be associated with increased BC mortality in the United States (US) [19]. Another US study found that higher individual, parental and life-course SES were positively associated with BC screening [20]. However, there is very limited research on the impact of parental and life-course education level on BC outcomes in developing countries, and none to our knowledge focused on cancer grade. Notably, the association of life-course SES factors such as education and BC grade is not well-defined for Nigerian patients despite disproportionately higher grade tumors observed in this population.

In this study, we will, for the first time, characterize the association between individual, parental, and life-course education and BC grade among Nigerian women. Findings will elucidate how population-level policies to address socioeconomic factors like education may be useful in improving BC prognoses in Nigeria.

## Materials and Methods

### Study design

The Mechanisms for Established and Novel Risk Factors for Breast Cancer in Nigerian Women (MEND) study has been previously described extensively [21]. Briefly, MEND enrolled patients at five hospital-based BC clinics in southwest Nigeria. An experienced research nurse described the study to patients visiting the clinic for a BC diagnosis. Upon presumed BC diagnosis (based on clinical evaluation, but pending pathological verification), patients were approached for participation in the study. Patients who expressed interest were then evaluated for eligibility, and the research nurse obtained written and verbal informed consent. Study participants completed a comprehensive interviewer-administered questionnaire that solicited information on sociodemographic characteristics, reproductive history, and health history, and physical measurements were taken. Routine biopsy was performed as part of the clinical standard of care, while additional research samples were obtained and sent for histopathology processing. In exchange for their participation, the participants received the supplies necessary for their clinical biopsy and a N500 telephone recharge card (approximately US $1.50). These study procedures were approved by the Institutional Review Boards of Duke University and the participating hospitals in Nigeria.

### Breast cancer diagnosis

BC diagnosis was verified in one of two possible ways – either a trained pathologist in Nigeria reviewed clinical biopsy samples, or research biopsy samples were shipped to the US for review by a trained US pathologist. BC grade from the Nigerian pathologists were reported on either the Nottingham or Scarff-Bloom-Richardson (SBR) scale. The Nottingham scale is a modification of the SBR grade scale, with both being similar three-level scales that are based on the tumor’s architectural pattern, nuclear atypia, and mitotic rate [22]. For samples processed in the US, grade was reported on the Nottingham scale. When grade data from both sources were available, the US grade information was used in the analysis. A sensitivity analysis considering only those samples with grade reported on the Nottingham scale was performed to ensure the validity of including data from both scales. Results were similar between both sets of data, therefore we defined grade based on either of the two scales and categorized into low (grades 1–2) and high (grade 3) for analysis.

### Study covariates

Demographic characteristics and clinical history variables included: age at diagnosis, marital status, number of comorbidities (such as hypertension, high lipids, high cholesterol, and diabetes), and whether participants had received a mammogram screening in the past two years. Participants were also asked to self-report any personal history of cancer outside of their BC diagnosis. Only those with no previous history of cancer were included in the present analysis since prior cancer diagnosis might be associated with a more aggressive subsequent tumor. Participant education level and parental education level was assessed based on self-reports by the participant and categorized as the following: low (primary school or less) or high (secondary school or greater). Secondary school and college education were combined into the “high” category due to low numbers among parents. Based on this categorization, life-course education was determined relative to each participant’s mother and father: stable high (if high participant education and high parental education), increasing (if high participant education and low parental education), decreasing (if low participant education and high parental education), or stable low (if low participant education and low parental education).

### Analytical approach

Descriptive statistics were used to characterize the study sample according to demographic and clinical characteristics and reported as frequencies and percentages. Further assessments of participant, parental, and life-course education variables were made by low/high-grade categorization. Differences in education variables (participant, parental, and life-course education) were tested using chi-square (χ^2^) tests. The association between each education variable and cancer grade was tested using logistic regression models with high-grade BC as the outcome, and results were presented as odds ratios and 95% confidence intervals (CI). Each measure (participant, parental and life-course education) was considered separately in a series of models: unadjusted, adjusted for age, and adjusted for age, number of comorbidities, marital status, and whether the participant had received a mammogram screening in the previous two years. In the models including participant and parental education, primary school or less was used as the reference group. In the models including life-course education, stable low was used as the reference group. SAS University Edition (Cary, North Carolina, United States) was used for all analyses and significance was set at α = 0.05.

## Results

**Figure 1:**
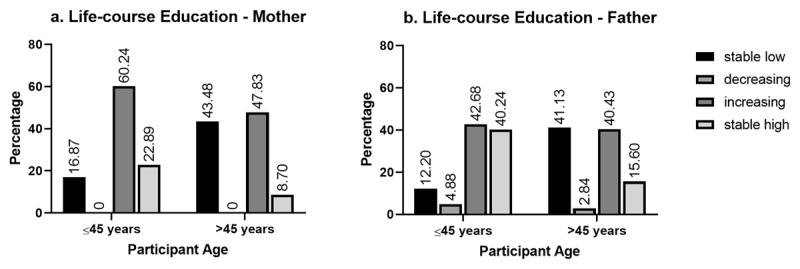
**Distribution of life-course education by participant age**. **a)** Distribution of life-course education categories relative to mother by age group. **b)** Distribution of life-course education categories relative to father by age group.

Most participants included in the analysis from the MEND study were between the ages of 40–49 years old (34%) and had low-grade BC (66%) (***Table 1***). Although about two-thirds (66%) of participants had a secondary school education or higher, most participants’ mothers and fathers had a primary school education or less (85% and 71%, respectively). Concerning mother, most participants had an increasing life-course education (52%), about one-third had stable low (33%), a small proportion had stable high (14%) and no participants had a decreasing life-course education. With respect to father, most participants also had an increasing life-course education (41%), almost one-third had stable low (30%), one-quarter had stable high (25%), and a low proportion had a decreasing life-course education (4%). When stratified by age, participants who were 45 years or younger were more likely to have a stable high life-course education compared to those older than 45 years old (***Figure 1***). A significantly higher proportion of participants who had high-grade BC, compared to low-grade cancer, had fathers with a primary school education or less (p = 0.04) (***Table 2***).

**Table 1:** Socio-demographic and Cancer Characteristics of Breast Cancer Cases Percentages may not add up to 100% due to missing values.


	OVERALL (%) N = 224 (100.00)

**Age**	

<40	41 (18.30)

40–49	76 (33.93)

50–59	58 (25.89)

60+	49 (21.88)

**Marital Status**	

Never Married	10 (4.46)

Married	157 (70.09)

Separated/Divorced	12 (5.36)

Widowed	45 (20.09)

**Mammogram Screening in Past 2 Years**	

Yes	26 (11.61)

No	197 (87.95)

**Comorbidities**	

None	17 (7.59)

1–2 Conditions	191 (85.27)

>2 Conditions	16 (7.14)

**Participant Education**	

Primary School or Less	76 (33.93)

Secondary/High School	73 (32.59)

College +	75 (33.48)

**Mother Education**	

Primary School or Less	190 (84.82)

Secondary/High School	18 (8.04)

College +	13 (5.80)

**Father Education**	

Primary School or Less	160 (71.43)

Secondary/High School	40 (17.86)

College +	23 (10.27)

**Life-course Education (Mother)**	

Stable Low	74 (33.04)

Decreasing	0 (0.00)

Increasing	116 (51.79)

Stable High	31 (13.84)

**Life-course Education (Father)**	

Stable Low	68 (30.36)

Decreasing	8 (3.57)

Increasing	92 (41.07)

Stable High	55 (24.55)

**Cancer Grade**	

1	15 (6.70)

2	132 (58.93)

3	77 (34.38)


**Table 2:** Education Level by Cancer Grade at Diagnosis.


	OVERALL	LOW-GRADE (1–2)	HIGH-GRADE (3)	P-VALUE*

	N = 224 (100.00)	N = 147 (65.63)	N = 77 (34.38)	

**Participant Education Level**				

Primary School or Less	76 (33.93)	48 (32.65)	28 (36.36)	0.5775

Secondary/High School +	148 (66.07)	99 (67.35)	49 (63.64)	

**Mother Education Level**				

Primary School or Less	190 (84.82)	123 (83.67)	67 (87.01)	0.3025

Secondary/High School +	31 (13.84)	23 (15.65)	8 (10.39)	

**Father Education Level**				

Primary School or Less	160 (71.43)	99 (67.35)	61 (79.22)	**0.0423**

Secondary/High School +	63 (28.13)	48 (32.65)	15 (19.48)	

**Life-course Education Level – Mother**				

Stable Low	74 (33.04)	48 (32.65)	26 (33.77)	0.5874

Decreasing	0 (0.00)	0 (0.00)	0 (0.00)	

Increasing	116 (51.79)	75 (51.02)	41 (53.25)	

Stable High	31 (13.84)	23 (15.65)	8 (10.39)	

**Life-course Education Level – Father**				

Stable Low	68 (30.36)	42 (28.57)	26 (33.77)	0.2478

Decreasing	8 (3.57)	6 (4.08)	2 (2.60)	

Increasing	92 (41.07)	57 (38.78)	35 (45.45)	

Stable High	55 (24.55)	42 (28.57)	13 (16.88)	


* Determined using Chi-Square tests Where applicable, missing values were not used to generate the p-value. Percentages may not add up to 100% due to missing values. Life-course education is defined based on participant and parent education level.

In fully adjusted multivariable regression models predicting high-grade BC (***Table 3***), having a father with a high versus low education was associated with 59% lower odds of having high-grade BC (aOR 0.41; 95% CI 0.20, 0.84). Stable high versus stable low life-course education with respect to father was associated with 64% lower odds of having high-grade BC (aOR 0.36; 95% CI 0.15, 0.87). The results for increasing life-course education (aOR 0.82; 95% Cl 0.40, 1.69) and decreasing life-course education (aOR 0.41; 95% CI 0.07, 2.46) with respect to father were not statistically significant. There were also no statistically significant results observed for participant education level (aOR 0.72; 95% CI 0.37, 1.36), mother education level (aOR 0.57; 95% CI 0.23, 1.38), and life-course education level with respect to mother (stable high aOR 0.51; 95% CI 0.19, 1.40, and increasing aOR 0.87; 95% CI 0.44, 1.71) across all the models.

**Table 3:** Multivariate Odds Ratios (OR) for High-Grade Diagnosis.


	UNADJUSTED	AGE	FULLY ADJUSTED

	OR (95% CI)	aOR (95% CI)^a^	aOR (95% CI)^b^

**Participant Education Level**			

Primary School or Less (Ref.)	–	–	–

Secondary/High School +	0.85 (0.48, 1.51)	0.68 (0.36, 1.29)	0.72 (0.37, 1.36)

**Mother Education Level**			

Primary School or Less (Ref.)	–	–	–

Secondary/High School +	0.64 (0.27, 1.51)	0.57 (0.24, 1.37)	0.57 (0.23, 1.38)

**Father Education Level**			

Primary School or Less (Ref.)	–	–	–

Secondary/High School +	**0.51 (0.26, 0.98)**	**0.43 (0.21, 0.85)**	**0.41 (0.20, 0.84)**

**Life-course Education Level – Mother**			

Stable Low (Ref.)	–	–	–

Decreasing	#	#	#

Increasing	1.01 (0.55, 1.86)	0.83 (0.43, 1.62)	0.87 (0.44, 1.71)

Stable High	0.64 (0.25, 1.64)	0.51 (0.19, 1.35)	0.51 (0.19, 1.40)

**Life-course Education Level – Father**			

Stable Low (Ref.)	–	–	–

Decreasing	0.54 (0.10, 2.87)	0.40 (0.07, 2.25)	0.41 (0.07, 2.46)

Increasing	0.99 (0.52, 1.89)	0.78 (0.39, 1.56)	0.82 (0.40, 1.69)

Stable High	0.50 (0.23, 1.10)	**0.36 (0.15, 0.84)**	**0.36 (0.15, 0.87)**


^a^ Adjusted for age. ^b^ Adjusted for age, mammogram screening, marital status, and comorbidities. OR = Odds Ratio. aOR = Adjusted Odds Ratio. # = Undefined. Bold indicates significance p-value ≤ 0.05. Life-course education is defined based on participant and parent education level.

## Discussion

In the present analysis of 224 female BC patients in the MEND study, we characterize the association between self-reported education (participant, parental, and life-course) and BC grade at diagnosis. We found that participants whose fathers were highly educated were more likely to have low-grade BC at diagnosis. Similarly, those with a stable high life-course education concerning father were more likely to have a low-grade cancer. Significant associations were not observed for individual participant education level, mother’s education level, and life-course education regarding mother.

It is well-recognized that higher SES is associated with lower cancer stage at clinical presentation and diagnosis and lower rates of mortality [11,12,13,14]. However, the association between SES and BC grade has not been as clearly reported, especially in low- and middle-income countries like Nigeria. One study in England found that socioeconomic deprivation was associated with higher BC grade at diagnosis [17]. Focusing specifically on education, an analysis of the National Cancer Database in the US found that living in more educationally-deprived areas is associated with higher BC grade [23]. This analysis was limited by a lack of data on individual-level educational attainment. Another study reported no significant association between education level and BC grade in a Turkish population [18]. However, unlike our analysis, this study was limited by a lack of data on covariates.

To date, most studies that report the association between SES and BC outcomes have focused only on the influence of patient SES in adulthood. As Williams et al. note, inadequate attention has been paid to understanding the effects of SES factors over the life-course [24]. In the context of mortality, one study in the US noted that father’s education, but not mother’s, is protective against deaths due to BC [25]. This is consistent with the findings of our current study, which indicates a better prognosis (through a lower grade cancer) for participants whose fathers are more highly educated. Similarly, a recent systematic review found some evidence that lower early life SES may be associated with increased BC mortality, but most of the studies included only used occupation to determine childhood SES, ultimately providing an incomplete picture of childhood effects [19]. Our analysis addresses this gap, reporting on the effects of education as a measure of early-life SES in a highly understudied population of Nigerian women. Most research that has included parental and life-course SES was conducted in populations from more developed countries, such as the US, Britain, and Sweden [25,26,27]. Similar research in developing countries, such as Nigeria, is limited. This is the first study to our knowledge to characterize the association between life-course education with respect to parents and BC grade in this population.

The observed association between BC grade and father’s education as well as life-course education may be a consequence of stressor accumulation over the life-course. In a Dutch population, researchers found that level of education is negatively associated with financial and psychological stress, and the absence of access to resources [28]. In turn, increased stress is related to increased cancer mortality and reduced BC survival [29]. Allostatic load (AL) is a term that encapsulates the biological dysregulation that may result from excessive exposure to repeated stressors. It has often been used in the context of racial disparities but may also offer insight on differences in health outcomes by SES and speaks to the disproportionate existence of high-grade BC subtypes among Black populations in the US [30,31]. Testing the association between education variables and BC subtype within this population may offer further insight. Furthermore, there is evidence that lower childhood socioeconomic positioning is associated with greater odds of adulthood obesity among women [32]. This may result from lower amounts of physical activity and more unhealthy food consumption due to a lack of resources [32,33]. In turn, obesity is associated with more aggressive, and higher-grade BC, potentially due to lower levels of circulating adiponectin [34,35]. Further research that considers the association between landmark biological features of obesity and BC grade is needed to shed light on these mechanisms. Along these lines, quality healthcare in Nigeria is costly and is often difficult for those of low SES to afford [36]. Provision of health care in early life may contribute to lower comorbidities like obesity that may increase the risk of high-grade BC.

Notably, this study found a significant association between father’s education, but not mother’s education, and BC grade at diagnosis. In the population studied, fathers had a secondary school or greater level of education at almost double the rate of mothers. This may be a consequence of a traditionally patriarchal society that valued the education of males over females, thus making father’s education a more robust predictor than mother’s and female participant’s education [37]. Interestingly, older participants were more likely to have a stable low life-course education (with reference to both mother and father), whereas younger participants were more likely to have a stable high life-course education. Given that all participants were female, this may speak to the increasing access to education for girls in Nigeria.

There are several limitations of this study that should be discussed. Firstly, BC grade was determined at several different hospitals by different pathologists, potentially introducing unstandardized or misclassified results. However, all grade information was reported according to either the Nottingham or SBR scales, and a sensitivity analysis was conducted to ensure the compatibility of these scales for the purposes of this analysis. Secondly, participants were asked to recall the education levels of their parents, which may have introduced some recall bias into the analysis. Thirdly, the observational nature of the study design makes it difficult to draw a causal relationship. However, this study has several notable strengths, including the involvement of participants from multiple hospital sites in Nigeria, the life-course approach, and the availability of data on relevant covariates.

## Conclusions

Higher father’s education and life-course education were associated with a lower likelihood of high BC grade at diagnosis. Early life SES may influence BC outcomes via accumulated stressors, well-established risk factors such as obesity, and access to quality and timely care. The results of this analysis suggest the importance of adopting a life-course approach towards understanding the relationship between SES and BC outcomes. We urge further research on this topic to inform population-level policies. Addressing childhood SES may be beneficial for improving BC outcomes in Nigeria.

## Data Accessibility Statement

The data that support the findings of the study are available from the corresponding author upon reasonable request.

## References

[1] Bray F, Ferlay J, Soerjomataram I, Siegel RL, Torre LA, Jemal A. Global cancer statistics 2018: GLOBOCAN estimates of incidence and mortality worldwide for 36 cancers in 185 countries. CA: a cancer journal for clinicians. 2018; 68(6): 394–424. DOI: 10.3322/caac.21492 30207593

[2] Forouzanfar MH, Foreman KJ, Delossantos AM, et al. Breast and cervical cancer in 187 countries between 1980 and 2010: A systematic analysis. Lancet (London, England). 2011; 378(9801): 1461–1484. DOI: 10.1016/S0140-6736(11)61351-2 21924486

[3] Azubuike SO, Muirhead C, Hayes L, McNally R. Rising global burden of breast cancer: The case of sub-Saharan Africa (with emphasis on Nigeria) and implications for regional development: a review. World Journal of Surgical Oncology. 2018; 16(1): 63. DOI: 10.1186/s12957-018-1345-2 29566711 PMC5863808

[4] Rakha EA, El-Sayed ME, Lee AH, et al. Prognostic significance of Nottingham histologic grade in invasive breast carcinoma. Journal of clinical oncology: Official journal of the American Society of Clinical Oncology. 2008; 26(19): 3153–3158. DOI: 10.1200/JCO.2007.15.5986 18490649

[5] Ugnat AM, Xie L, Morriss J, Semenciw R, Mao Y. Survival of women with breast cancer in Ottawa, Canada: Variation with age, stage, histology, grade and treatment. British journal of cancer. 2004; 90(6): 1138–1143. DOI: 10.1038/sj.bjc.6601662 15026792 PMC2409653

[6] Adisa CA, Eleweke N, Alfred AA, et al. Biology of breast cancer in Nigerian women: A pilot study. Annals of African medicine. 2012; 11(3): 169–175. DOI: 10.4103/1596-3519.96880 22684136

[7] Ikpatt OF, Kuopio T, Ndoma-Egba R, Collan Y. Breast cancer in Nigeria and Finland: Epidemiological, clinical and histological comparison. Anticancer research. 2002; 22(5): 3005–3012. 12530033

[8] Dewalt DA, Berkman ND, Sheridan S, Lohr KN, Pignone MP. Literacy and health outcomes: A systematic review of the literature. Journal of general internal medicine. 2004; 19(12): 1228–1239. DOI: 10.1111/j.1525-1497.2004.40153.x 15610334 PMC1492599

[9] Hahn RA, Truman BI. Education Improves Public Health and Promotes Health Equity. International journal of health services: Planning, administration, evaluation. 2015; 45(4): 657–678. DOI: 10.1177/0020731415585986 PMC4691207 25995305

[10] Albano JD, Ward E, Jemal A, et al. Cancer mortality in the United States by education level and race. Journal of the National Cancer Institute. 2007; 99(18): 1384–1394. DOI: 10.1093/jnci/djm127 17848670

[11] Liu Y, Zhang J, Huang R, et al. Influence of occupation and education level on breast cancer stage at diagnosis, and treatment options in China: A nationwide, multicenter 10-year epidemiological study. Medicine. 2017; 96(15): e6641. DOI: 10.1097/MD.0000000000006641 28403116 PMC5403113

[12] Mathew A, George PS, Ramadas K, et al. Sociodemographic Factors and Stage of Cancer at Diagnosis: A Population-Based Study in South India. Journal of Global Oncology. 2019; 5: 1–10. DOI: 10.1200/JGO.18.00160 PMC6690651 31322993

[13] Jedy-Agba E, McCormack V, Olaomi O, et al. Determinants of stage at diagnosis of breast cancer in Nigerian women: sociodemographic, breast cancer awareness, health care access and clinical factors. Cancer causes & Control: CCC. 2017; 28(7): 685–697. DOI: 10.1007/s10552-017-0894-y 28447308 PMC5492222

[14] Mohaghegh P, Yavari P, Akbari ME, Abadi A, Ahmadi F. Associations of demographic and socioeconomic factors with stage at diagnosis of breast cancer. Asian Pacific Journal of Cancer Prevention: APJCP. 2015; 16(4): 1627–1631. DOI: 10.7314/APJCP.2015.16.4.1627 25743843

[15] Nuche-Berenguer B, Sakellariou D. Socioeconomic determinants of cancer screening utilisation in Latin America: A systematic review. PloS one. 2019; 14(11): e0225667. DOI: 10.1371/journal.pone.0225667 31765426 PMC6876872

[16] Willems B, Bracke P. The education gradient in cancer screening participation: A consistent phenomenon across Europe? International journal of public health. 2018; 63(1): 93–103. DOI: 10.1007/s00038-017-1045-7 29063122

[17] Taylor A, Cheng KK. Social deprivation and breast cancer. Journal of Public Health Medicine. 2003; 25(3): 228–233. DOI: 10.1093/pubmed/fdg072 14575198

[18] Sarici F, Babacan T, Buyukhatipoglu H, et al. Correlation of educational status and clinicopathological characteristics of breast cancer: A single center experience. Journal of BUON: official journal of the Balkan Union of Oncology. 2016; 21(4): 826–831. 27685902

[19] Akinyemiju TF, Demb J, Izano MA, et al. The association of early life socioeconomic position on breast cancer incidence and mortality: A systematic review. International Journal of Public Health. 2018; 63(7): 787–797. DOI: 10.1007/s00038-017-1060-8 29197969 PMC5984656

[20] Akinyemiju T, Ogunsina K, Sakhuja S, Ogbhodo V, Braithwaite D. Life-course socioeconomic status and breast and cervical cancer screening: Analysis of the WHO’s Study on Global Ageing and Adult Health (SAGE). BMJ open. 2016; 6(11): e012753. DOI: 10.1136/bmjopen-2016-012753 PMC5129035 27881528

[21] Akinyemiju T, Salako O, Daramola A, et al. Collaborative Molecular Epidemiology Study of Metabolic Dysregulation, DNA Methylation, and Breast Cancer Risk Among Nigerian Women: MEND Study Objectives and Design. Journal of Global Oncology. 2019; 5: 1–9. DOI: 10.1200/JGO.18.00226 PMC6613666 31194608

[22] Elston CW, Ellis IO. Pathological prognostic factors in breast cancer. I. The value of histological grade in breast cancer: experience from a large study with long-term follow-up. Histopathology. 1991; 19(5): 403–410. DOI: 10.1111/j.1365-2559.1991.tb00229.x 1757079

[23] DeSantis C, Jemal A, Ward E. Disparities in breast cancer prognostic factors by race, insurance status, and education. Cancer Causes & Control: CCC. 2010; 21(9): 1445–1450. DOI: 10.1007/s10552-010-9572-z 20506039

[24] Williams DR, Mohammed SA, Shields AE. Understanding and effectively addressing breast cancer in African American women: Unpacking the social context. Cancer. 2016; 122(14): 2138–2149. DOI: 10.1002/cncr.29935 26930024 PMC5588632

[25] Pudrovska T, Anikputa B. The role of early-life socioeconomic status in breast cancer incidence and mortality: Unraveling life course mechanisms. Journal of Aging and Health. 2012; 24(2): 323–344. DOI: 10.1177/0898264311422744 21956096 PMC3428234

[26] Power C, Hyppönen E, Smith GD. Socioeconomic position in childhood and early adult life and risk of mortality: A prospective study of the mothers of the 1958 British birth cohort. American Journal of Public Health. 2005; 95(8): 1396–1402. DOI: 10.2105/AJPH.2004.047340 15985645 PMC1449372

[27] Lawlor DA, Sterne JA, Tynelius P, Davey Smith G, Rasmussen F. Association of childhood socioeconomic position with cause-specific mortality in a prospective record linkage study of 1,839,384 individuals. American Journal of Epidemiology. 2006; 164(9): 907–915. DOI: 10.1093/aje/kwj319 16987923

[28] Mulder BC, de Bruin M, Schreurs H, van Ameijden EJ, van Woerkum CM. Stressors and resources mediate the association of socioeconomic position with health behaviours. BMC Public Health. 2011; 11: 798. DOI: 10.1186/1471-2458-11-798 21991933 PMC3205066

[29] Chida Y, Hamer M, Wardle J, Steptoe A. Do stress-related psychosocial factors contribute to cancer incidence and survival? Nature Clinical Practice Oncology. 2008; 5(8): 466–475. DOI: 10.1038/ncponc1134 18493231

[30] Geronimus AT, Hicken M, Keene D, Bound J. “Weathering” and age patterns of allostatic load scores among blacks and whites in the United States. American Journal of Public Health. 2006; 96(5): 826–833. DOI: 10.2105/AJPH.2004.060749 16380565 PMC1470581

[31] Furberg H, Millikan R, Dressler L, Newman B, Geradts J. Tumor characteristics in African American and white women. Breast cancer research and treatment. 2001; 68(1): 33–43. DOI: 10.1023/A:1017994726207 11678307

[32] Senese LC, Almeida ND, Fath AK, Smith BT, Loucks EB. Associations between childhood socioeconomic position and adulthood obesity. Epidemiologic reviews. 2009; 31: 21–51. DOI: 10.1093/epirev/mxp006 19648176 PMC2873329

[33] Hanson MD, Chen E. Socioeconomic status and health behaviors in adolescence: A review of the literature. Journal of Behavioral Medicine. 2007; 30(3): 263–285. DOI: 10.1007/s10865-007-9098-3 17514418

[34] Vona-Davis L, Rose DP. The influence of socioeconomic disparities on breast cancer tumor biology and prognosis: A review. Journal of Women’s Health (2002). 2009; 18(6): 883–893. DOI: 10.1089/jwh.2008.1127 19514831

[35] Engin A. Obesity-associated Breast Cancer: Analysis of risk factors. Advances in Experimental Medicine and Biology. 2017; 960: 571–606. DOI: 10.1007/978-3-319-48382-5_25 28585217

[36] Chukwudozie A. Inequalities in Health: The Role of Health Insurance in Nigeria. J Public Health Afr. 2015; 6(1): 512. DOI: 10.4081/jphia.2015.512 28299138 PMC5349265

[37] Ajala TJIJoG, Studies W. Social construction of gender roles and women’s poverty in African societies: The Case of the Nigerian Woman. 2016; 4(2): 1–10. DOI: 10.15640/ijgws.v4n2a1

